# Adhesion patterns of commensal and pathogenic *Escherichia coli* from humans and wild animals on human and porcine epithelial cell lines

**DOI:** 10.1186/1757-4749-5-31

**Published:** 2013-11-04

**Authors:** Ulrike Frömmel, Alexander Böhm, Jörg Nitschke, Jörg Weinreich, Julia Groß, Stefan Rödiger, Thomas Wex, Hermann Ansorge, Olaf Zinke, Christian Schröder, Dirk Roggenbuck, Peter Schierack

**Affiliations:** 1 Brandenburg Technical University Cottbus/Senftenberg, Faculty of Natural Sciences, Großenhainer Str. 57, D-01968, Senftenberg, Germany; 2 Otto-von-Guericke University, Magdeburg, Germany; 3 Senckenberg Museum, Görlitz, Germany; 4 Museum der Westlausitz, Kamenz, Germany; 5 GA Generic Assays GmbH, Dahlewitz, Germany

**Keywords:** *E. coli*, Adhesion patterns, Virulence-associated genes, Pathotypes, IPEC-J2, PK-15, Caco-2, 5637, HEp-2

## Abstract

**Background:**

Different strategies of colonization or infection by *E*. *coli* result in formation of certain adhesion patterns which help also in classifying intestinal *E*. *coli* into pathotypes. Little is known about adhesion patterns and host- and tissue adaption of commensal *E*. *coli* and about *E*. *coli* originating in clinically healthy hosts carrying pathotype-specific virulence-associated genes.

**Findings:**

Adhesion pattern of *E*. *coli* (n = 282) from humans and from 18 animal species were verified on intestinal human Caco-2 and porcine IPEC-J2 cells and, furthermore, for comparison on human urinary bladder 5637, porcine kidney PK-15 epithelial and HEp-2 cells. The analysis was carried out on 150,000 images of adhesion assays.

Adhesion patterns were very diverse; 88 isolates were completely non-adherent, whereas 194 adhered to at least one cell line with the dominant adhesion patterns “diffusely distributed” and “microcolony formation”. Adhesion patterns “chains” and “clumps” were also visible. Chain formation was mediated by the presence of epithelial cells. Clump formation was very specific on only the 5637 cell line. All enteropathogenic (*eae*^+^) *E*. *coli* (EPEC; n = 14) were able to form microcolonies which was cell line specific for each isolate. Most EPEC formed microcolonies on intestinal IPEC-J2 and Caco-2 but several also on urinary tract cells. Shigatoxin-producing (*stx*^+^) *E*. *coli* (n = 10) showed no specific adhesion patterns.

**Conclusions:**

*E*. *coli* isolates were highly diverse. Commensal and pathogenic isolates can adhere in various forms, including diffuse distribution, microcolonies, chains and clumps. Microcolony formation seems to be a global adhesion strategy also for commensal *E*. *coli*.

## Findings

### Background

The occurrence of bacterial virulence factors coded by virulence-associated genes (VAGs) indicates different host infection mechanisms and adhesion patterns, and can be used to define intestinal *E*. *coli* pathotypes [1]. Pathotype-specific VAGs frequently detected in *E*. *coli* from diarrheic hosts can also be found in *E*. *coli* from clinically healthy hosts [2,3]. It is neither known if such isolates display adhesion patterns similar to their pathogenic counterparts nor whether their adhesion or infection patterns are host-specific and tissue-specific. Moreover, no information is available regarding host- or tissue-specific adhesion patterns of *E*. *coli* without VAGs (commensals). We analyzed adhesion patterns of 282 intestinal *E*. *coli* isolates on four epithelial cell lines. Due to such a large amount of isolates, we created an automatic imaging method based on fluorescence microscopy.

## Results

### Adhesion patterns of *E. coli*

Of 282 *E*. *coli*, 28 isolates (9.9%) carried at least one VAG which defined 14 enteropathogenic (EPEC), 10 Shigatoxin-producing (STEC), 3 enterotoxigenic (ETEC) and 1 *daa*D^+^ diffusely adherent *E*. *coli* (DAEC) (Table 1; [4]). Two EPEC were typical (tEPEC, pEAF^+^) and 12 were atypical EPEC (aEPEC, pEAF^-^). Most isolates (68.8%) adhered to at least one cell line but 31.2% of isolates were non-adherent. Adhesion and pattern formation was most present on Caco-2, followed by IPEC-J2, 5637 and PK-15 cells (Table 2 and 3).

**Table 1:** **Species of isolated *E. coli and detected intestinal E. coli pathotypes***

**Species**		**Number of *E*. *coli *** **isolates**	**aEPEC**	**tEPEC**	**STEC**	**ETEC**	**DAEC**
**Mammals**							
*Homo sapiens*	Human	19^(a)^	-	-	1	-	1
*Sus scrofa domestica*	Domestic pig	22^(b)^	-	-	-	1	-
*Capreolus capreolus*	Roe deer	23^(c,d)^	-	-	9	1	-
*Erinaceus europaeus*	European hedgehog	22^(c)^	-	1	-	-	-
*Lepus europaeus*	European hare	8^(c)^	3	-	-	-	-
*Lutra lutra*	European otter	7^(c)^	-	-	-	-	-
*Martes sp*.	Marten	19^(c)^	3	-	-	-	-
*Meles meles*	European badger	7^(c)^	1	-	-	-	-
*Mus musculus*	House mouse	9^(c)^	-	-	-	-	-
*Oryctolagus cuniculus*	European rabbit	6^(c)^	-	1	-	-	-
*Procyon lotor*	Raccoon	22^(c)^	2	-	-	-	-
*Rattus norvegicus*	Brown rat	4^(c)^	-	-	-	-	-
*Sciurus vulgaris*	Red squirrel	17^(c)^	2	-	-	-	-
*Sus scrofa*	Wild boar	22^(c,d)^	-	-	-	-	-
*Vulpes vulpes*	Red fox	21^(c)^	1	-	-	1	-
**Birds**							
*Accipiter nisus*	Eurasian sparrowhawk	13^(c)^	-	-	-	-	-
*Asio otus*	Long-eared owl	5^(c)^	-	-	-	-	-
*Buteo buteo*	Common buzzard	14^(c)^	-	-	-	-	-
*Turdus merula*	Common blackbird	22^(c)^	-	-	-	-	-

^(a)^Isolates sampled by Thomas Wex, Otto-von-Guericke University, Magdeburg, Germany; ^(b)^Isolates collected from eighteen different pig production units in Eastern Germany in the years 2009/2010;

^(c)^Isolates collected from dead animals in the LausitzLusatia), a region in southeastern Germany, which were directly collected as accident victims or which were delivered to the Senckenberg MuseumGörlitz, Germany) and the Museum der WestlausitzKamenz, Germany) between 2007 and 2011;

^(d)^Isolates sampled during several hunts between 2007 and 2010.

**Table 2:** ***E***. ***coli *adhesion patterns**: **cell line**, **tissue**, **host cell specificity**

**Adhesion pattern specificity**	**Cell lines**	**Diffusely distributed**	**Micro-colonies**	**Chains**	**Various pattern**
Cell line	Caco-2	28	6	1	-
	IPEC-J2	12	3	-	-
	5637	10	-	-	-
	PK-15	1	-	-	-
Tissue	Intestinal epithelium (Caco-2, IPEC-J2)	16	9	-	1
	Urinary epithelium (5637, PK-15)	1	-	-	-
Host cell	Human (Caco-2, 5637)	9	8	-	1
	Porcine (IPEC-J2, PK-15)	3	3	-	-
Unspecific	All cell lines	8	20	3	12
	Other groups of cell lines	21	8	2	8

Classified are isolates which adhered with a distinct pattern (diffusely distributed, microcolonies, chains) to at least one cell line (n = 172), and isolates with different adhesion patterns to the four cell lines (various pattern, n = 22). Non-adherent isolates (n = 88) were excluded. Most distinct pattern-forming isolates were diffusely distributed on at least one cell line (n = 109) followed by microcolony forming isolates (n = 57). “Other groups of cell lines” includes isolates with adhesion patterns on the following cell lines: 1) Caco-2 and 5637 and IPEC-J2; 2) Caco-2 and 5637 and PK-15; 3) Caco-2 and IPEC-J2 and PK-15; 4) 5637 and IPEC-J2 and PK-15; 5) Caco-2 and PK-15; 6) 5637 and IPEC-J2.

**Table 3:** **Number of *E. coli *** **isolates from human and 18 animal species and its adhesion patterns on Caco-2, IPEC-J2, 5637 and PK-15**

	**Human**	**Domestic pig**	**Roe deer**	**Europ. hedgehog**	**Europ. hare**	**Europ. otter**	**Mar ten**	**Europ. badger**	**House mouse**	**Europ. rabbit**	**Rac coon**	**Brown rat**	**Red squirrel**	**Wild boar**	**Red fox**	**Eurasian sparrow hawk**	**Long-eared owl**	**Common buzzard**	**Common blackbird**	**sum**
Isolates	19	22	23	22	8	7	19	7	9	6	22	4	17	22	21	13	5	14	22	282
**Caco**-**2**																				
Microcolonies	5	4	3	6	4	-	6	2	-	1	6	2	8	1	4	-	-	2	8	62
Chains	-	-	2	-	-	-	3	-	-	-	-	-	-	2	-	-	-	1	1	9
Distributed	5	5	5	1	3	5	2	4	3	4	8	1	8	1	3	8	3	7	8	84
Non-adherent	9	13	13	15	1	2	8	1	6	1	8	1	1	18	14	5	2	4	5	127
**IPEC**-**J2**																				
Microcolonies	3	3	2	6	3	-	7	2	-	2	2	2	5	-	6	1	-	1	7	52
Chains	-	-	-	1	-	-	-	-	-	-	-	-	-	2	-	-	-	1	1	5
Distributed	7	4	3	5	-	2	7	2	3	1	1	-	3	1	-	3	1	3	9	55
Non-adherent	9	15	18	10	5	5	5	3	6	3	19	2	9	19	15	9	4	9	5	170
**5637**																				
Microcolonies	1	2	4	5	1	-	5	1	-	1	4	2	4	1	3	-	-	1	4	39
Chains	-	-	1	-	-	-	1	-	-	-	-	-	-	2	-	-	-	-	2	6
Clumps	1	-	-	-	-	-	-	-	-	-	-	-	1	-	-	-	-	-	-	2
Distributed	6	5	2	2	2	2	3	-	-	-	3	-	3	5	3	2	1	2	2	43
Non-adherent	11	15	16	15	5	5	10	6	9	5	15	2	9	14	15	11	4	11	14	192
**PK**-**15**																				
Microcolonies	6	2	3	2	2	-	4	1	-	2	2	2	2	-	4	-	-	1	6	39
Chains	-	-	1	-	-	-	1	-	-	-	-	-	-	2	-	-	-	1	1	6
Distributed	2	3	1	1	1	-	3	1	1	2	-	1	3	1	4	3	1	5	1	34
Non-adherent	11	17	18	19	5	7	11	5	8	2	20	1	12	19	13	10	4	7	14	203

Distributed = diffuse adherent and diffusely distributed.

**Figure 1:**
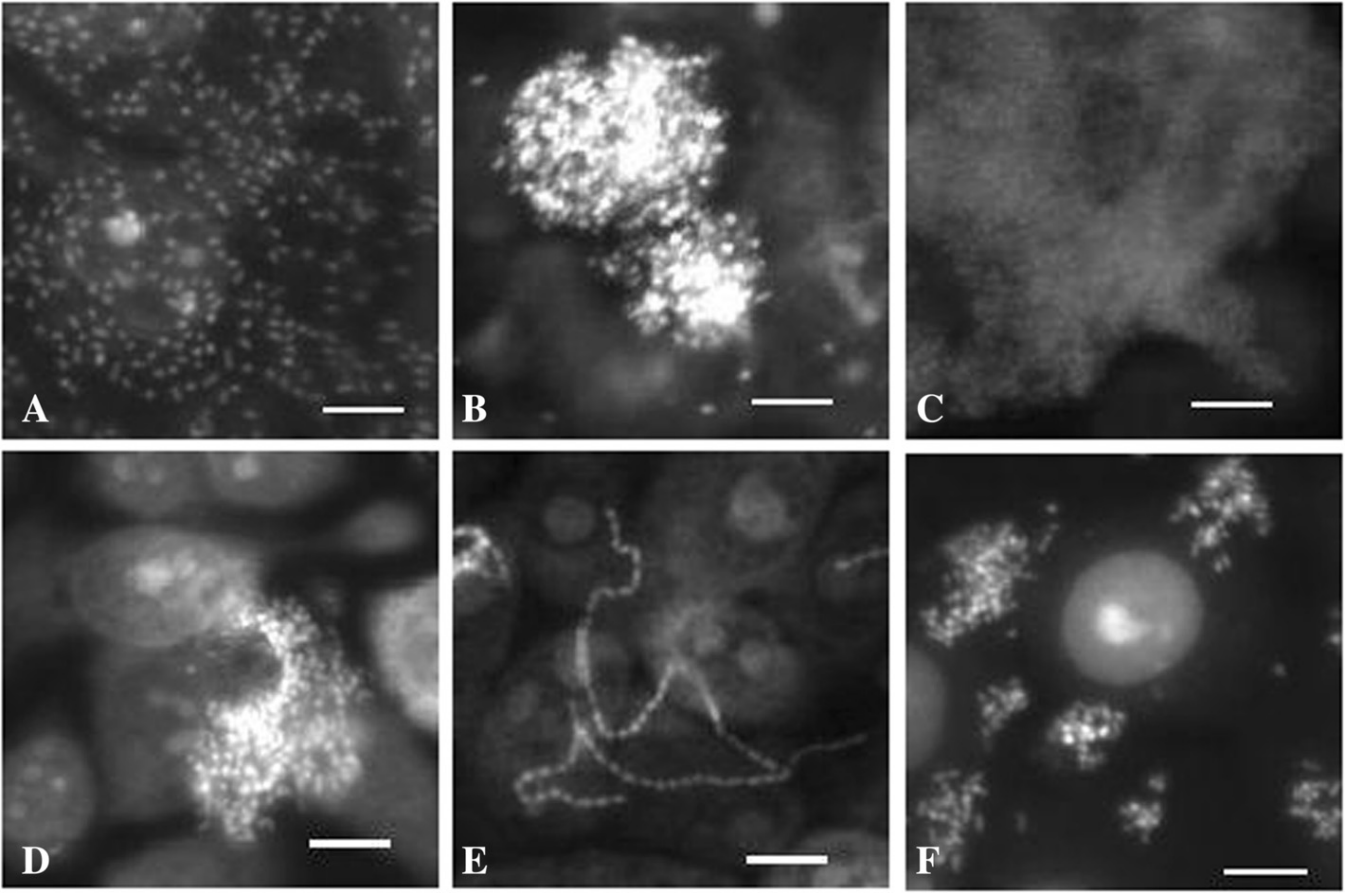
***E. coli *adhesion patterns of commensal *E. coli *and tEPEC.** Fluorescent images of epithelial cells and adherent bacteria which were stained with propidium iodide are depicted. **A)***E*. *coli* isolate from a domestic pig adhering diffusely distributed to IPEC-J2 cells. **B)***E*. *coli* isolate from a European hare forming microcolonies on Caco-2 cells. **C)***E*. *coli* isolate from a human forming clumps on 5637 cells. **D)***E*. *coli* isolate from a common blackbird forming microcolonies on 5637 cells. **E)***E*. *coli* isolate from a wild boar forming chains on PK-15 cells. **F)** tEPEC isolate from a European hedgehog forming microcolonies on 5637 cells. Scale: 10 μm.

**Figure 2:**
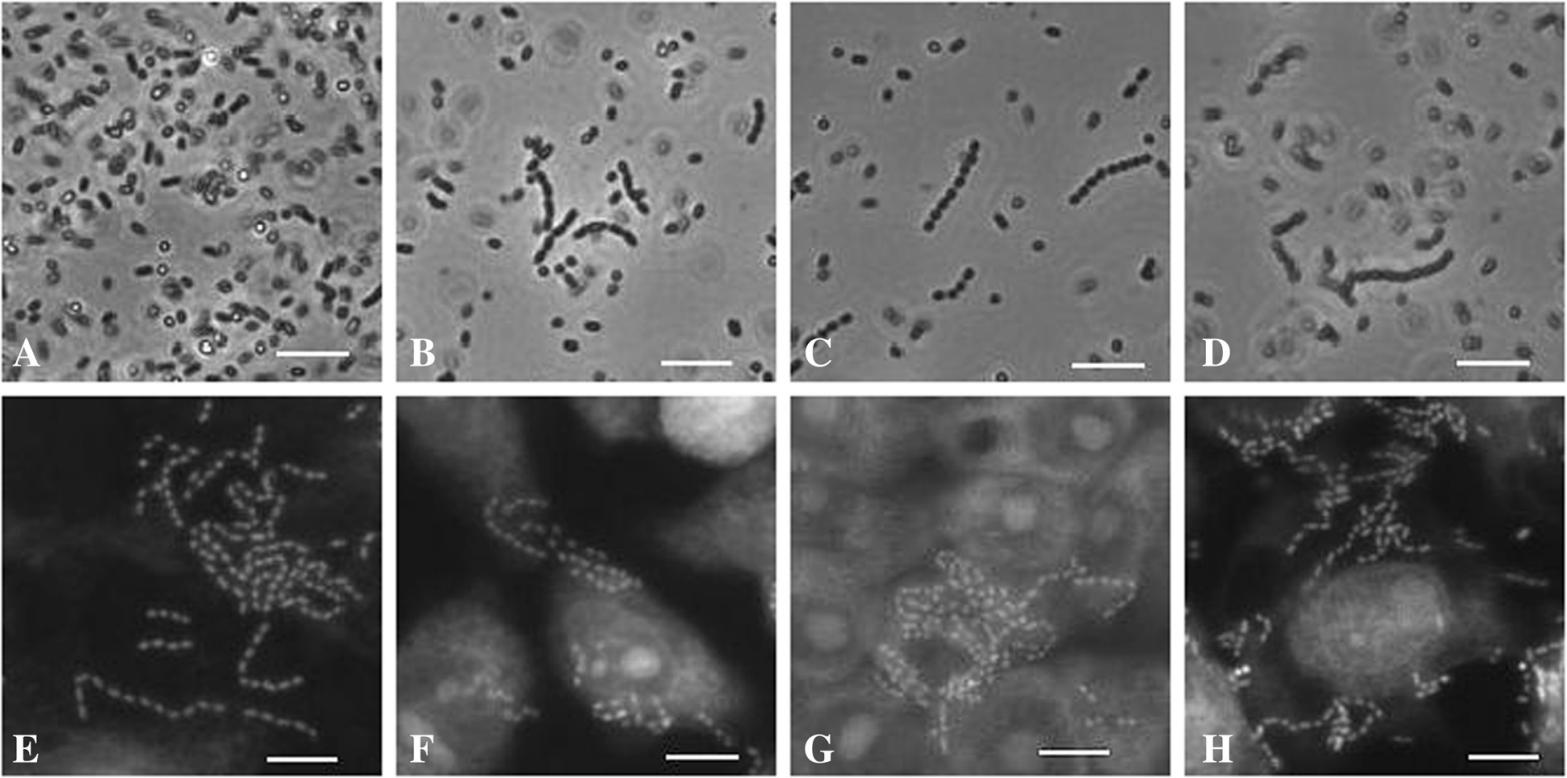
**Chain formation of one *E. coli *isolate from a common blackbird.** Fluorescence images were taken after incubation in LB medium (overnight), cell culture media (four hours) and after incubation on epithelial cells (four hours). Chain formation is induced by cell culture media, and stimulated by epithelial cells. Isolate in **A)** LB medium: no chains, **B)** Caco-2 cell culture medium, **C)** 5637 cell culture medium, **D)** IPEC-J2 and PK-15 cell culture medium. Isolate after four-hour adhesion assay with cell culture media on **E)** Caco-2, **F)** 5637, **G)** PK-15 cells. **H)** Isolate after four-hour adhesion assay with LB media on IPEC-J2 cells. **A-D)** Phase contrast microscopy. **E-H)** Fluorescence microscopy. Scale: 10 μm.

We identified four adhesion pattern of all *E*. *coli*: (i) diffusely distributed single bacteria; (ii) microcolonies; (iii) chains; and (iv) clumps (Figure 1). Isolates formed one adhesion pattern on one cell line only (cell line-specific), on all cell lines (both, host cell-unspecific and tissue-unspecific), on urinary tract or intestinal cells (tissue-specific) or on human or porcine cells (host cell-specific) (Table 2). There was no species-specific adhesion pattern which means that *E*. *coli* isolates from one animal host displayed diverse adhesion patterns on one cell line (Table 3). Two isolates formed clumps on cell line 5637 (Figure 1C) but were distributed diffusely or formed microcolonies on other cell lines. Chain formation could be identified in eleven isolates and was unspecific. Chain formation was inducible by cell culture medium as well as by the epithelial cells themselves (Figure 2).

### Associations between pathotypes and adhesion patterns

**Figure 3:**
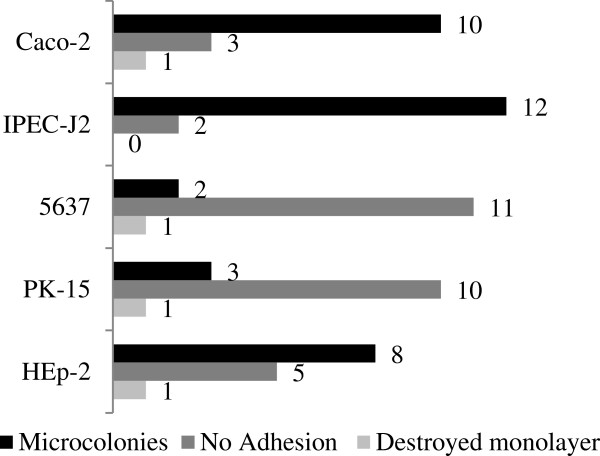
**Adhesion pattern formation of 14 *eae*A**^**+ **^***E. coli *isolates on four epithelial cells and on HEp-2 cells after six-hour incubation.** Microcolonies were formed on IPEC-J2 (10 aEPEC, 2 tEPEC), Caco-2 (10 aEPEC, no tEPEC), HEp-2 (7 aEPEC, 1 tEPEC), PK-15 (2 aEPEC, 1 tEPEC) and 5637 cells (2 aEPEC, no tEPEC).

EPEC: We tested the microcolony formation of EPEC on intestinal and urinary tract cells and on the model cell line HEp-2. All *eae*A^+^ isolates formed microcolonies on at least two cell lines but no isolate formed microcolonies on all cell lines. aEPEC formed microcolonies on IPEC-J2 (10 aEPEC), Caco-2 (10), HEp-2 (7), PK-15 (2) and 5637 cells (2) (Figure 3) STEC: All STEC were non-adherent to any tested cell line with the exception of 6 STEC; 5 were low adherent on Caco-2 cells; one was low adherent on human cells ETEC: A consistent adhesion pattern was not observed for the three ETEC *daa*D^+^ DAEC. The one human *daa*D^+^ DAEC adhered diffusely to human intestinal and urinary tract and also to HEp-2 cells but did not adhere to porcine cells.

## Discussion and conclusion

In contrast to conventional adhesion studies which include relatively low numbers of isolates and cell lines, the present study included 282 *E*. *coli* isolates and their adhesion patterns on four cell lines. To handle such large sample numbers, assays were adapted to the 96-well cell culture format and to the fluorescence microscopy-based technology [5].

### Adhesion patterns of commensals

The expression of different bacterial adhesins binding on host cell receptors drives bacterial tissue and host specificity [6,7]. We demonstrated that most isolates had different adhesion patterns on different cell lines. However, there were also isolates which had one adhesion pattern on all cell lines. This indicates the broad spectrum of adhesin-receptor interactions of *E*. *coli* in the intestinal and urinary tract. Microcolony formation was more unspecific than diffuse distribution, thus indicating that microcolony formation is less dependent on cell type specific receptors or response.

It is well known that EPEC forms microcolonies. This has also been demonstrated in the case of our 14 *eae*A^+^ isolates. However, there were other 61 isolates which also formed microcolonies but were intimin-negative according to our PCR which was able to detect all known intimin types. This shows that microcolony formation is more of a global adhesion and colonization strategy which ensues resistance to environmental or host immunological stresses [8,9].

*E*. *coli* chain formation was just recently published by Gioppo *et al*. (2000) and Vejborg and Klemm (2009) [10,11]. Our isolates did not form chains in pure LB medium but in cell culture media or on epithelial cells which is in contrast to these previous studies [11]. This, and the fact that chains were longer on cells than chains in cell culture media alone, showed that epithelial cells mediated *E*. *coli* chain formation.

Two isolates of our study exhibited a very unique adhesion pattern on 5637 cells only. They formed large clumps of several thousand bacteria which was not been described so far. We assume these two isolates have a high potential for biofilm formation.

### Adhesion patterns of intestinal pathogens

EPEC: All 14 intimin-positive isolates formed microcolonies on at least two cell lines verifying that these isolates are indeed EPEC. Host cell-unspecific and tissue-unspecific microcolony formation of some isolates showed that microcolony formation can be a universal infection mechanism. Other isolates showed tissue or host tropism which often depends on different intimin subtypes. Interestingly, several EPEC formed microcolonies on urinary tract epithelial cells, a fact which has not previously been recorded and which might contribute to *E*. *coli* urinary tract infections. Finally, IPEC-J2 turned out to be a better model cell line for the verification of the EPEC phenotype than HEp-2 cells and may represent an additional valuable diagnostic tool. STEC: In general, the adhesion of STEC isolates to any tested cell line was very low, supporting the non-adhesive infection mechanism of STEC [12]. ETEC and DAEC: As we detected only 3 ETEC and only 1 DAEC, we have not gone into any discussion about adhesion pattern specificity here.

In conclusion, adhesion patterns as well as host and tissue specificity varied broadly between isolates. Since diffuse adherence was also detected for many commensal *E*. *coli*, any characterization of pathogenic DAEC based solely on the determination of pattern formation would appear to be unsuitable. Since many commensal isolates also formed microcolonies, this adhesion pattern must be recognized as a global colonization strategy.

## Methods

### Bacterial isolates

Isolates are listed in Table 1. Identification and confirmation of *E*. *coli* is extensively described elsewhere [4,13-15]. Hemolytic isolates were excluded from analysis as they destroy cell monolayers. For human samples, sample collection was performed by individuals themselves using sterile collection tubes. The samples represented the negative controls of a clinical study. Participants were prospectively informed about the potential usage of redundant stool samples for research purpose; no written informed consent was obtained from patients. All associated information relating to these samples was anonymous. This study was approved by the Ministry of Environment, Health and Consumer Protection of the Federal State of Brandenburg, Germany (V3-2347-8-39-1-2011).

### Multiplex PCR assay

*E*. *coli* pathotyping based on the occurrence of VAGs: tEPEC (*eae*A^+^ pEAF^+^); aEPEC (*eae*A^+^ pEAF^-^); STEC (*stx*1 and/or *stx*2); enterohemorrhagic *E*. *coli*/EHEC (*stx*1 and/or *stx*2 and *eae*A); ETEC (*elt*B and/or *est*1 and/or *est*2); DAEC (*daa*D); enteroaggregative *E*. *coli*/EAEC (*agg*R); enteroinvasive *E*. *coli*/EIEC (*ipa*H); and commensals (no VAGs). The relevant multiplex PCR and PCR results were already described [4]. The pEAF-PCR was prepared according to Franke *et al*. 1994 [16]. Primers are listed in Table 4.

**Table 4:** **Primers for VAGs of *E***. ***coli***

**Genes**		**Primer and probe sequences**	**Fragment length**	**Accession**	**Source of primer**
		**(5′ 3 f: forward; r: reverse)**			
**Multiplex PCR**					
*ipa*H	f	GTTCCTTGACCGCCTTTCCGATACCGTC	619	M32063	[17]
	r	GCCGGTCAGCCACCCTCTGAGAGTAC			
*daa*D	f	TGAACGGGAGTATAAGGAAGATG	371	AY525531.1	[17]
	r	GTCCGCCATCACATCAAAA			
*elt*B	f	TCTCTATGTGCATACGGAGC	322	EU113252.1	[17]
	r	CCATACTGATTGCCGCAAT			
*eae*A	f	CCTGGTTACAACATTATGGAACG	287	AJ308550.1	[4]
	r	TGAAATAGTCTCGCCAGTATTCG			
*stx*2	f	GGCACTGTCTGAAACTGCTCC	255	FN252459	[17]
	r	TCGCCAGTTATCTGACATTCTG			
*agg*R	f	CGTAAGCCGGGTATGAAAGA	188	Z32523.1	[4]
	r	GCCAGTTCAGAAGCAGGAAC			
*est*1a	f	TTTCCCCTCTTTTAGTCAGTCAA	159	M25607.1 AY342057.1	[17]
	r	GCAGGATTACAACACAATTCACAGCAG			
*stx*1	f	CTGGATTTAATGTCGCATAGTG	150	HM367099.1	[17]
	r	AGAACGCCCACTGAGATCATC			
*est*2	f	CTATTGCTACAAATGCCTATGC	126	M35586.1	[4]
	r	CTCCAGCAGTACCATCTCTA			[18]
**Single PCR**					
pEAF	f	CAGGGTAAAAGAAAGATGATAA	397	X76137.1	[16]
	r	TATGGGGACCATGTATTATCA			

### *E. coli* adhesion assays

All cells were grown and adhesion assays were carried out as previously described [4,19]. *E*. *coli* were grown overnight to an OD_600_ of 0.8-1.2. Cells were inoculated with an infection dose of 62,500 bacteria per mm^2^ of a monolayer using a conversion of 3x10^8^ bacteria/mL/OD_600_. After four hour incubation and washing with 1xPBS, cells and adherent bacteria were fixed with 4% paraformaldehyde. Cells and bacteria were stained with propidium iodide (10 μg/mL in ddH_2_O) and analyzed. All tests were repeated at least three times in triplicates. To verify the EPEC phenotype, *eae*A^+^ isolates were carried out with six hours incubation time on epithelial cells including one additional washing step after three hours. The term “diffuse adherent” was used exclusively to refer to *daa*D^+^ DAEC. A similar pattern of isolates not containing *daa*D was defined as “diffuse distributed”.

### VideoScan/Aklides­: fluorescence imaging technology

The VideoScan technology implemented in the commercially available Aklides System (Medipan GmbH, Dahlewitz/Berlin, Germany) is a versatile fluorescence microscope imaging technology which can be used to analyze fluorescent objects [4,5,20-22]. The VideoScan/Aklides instrument automatically aligned itself to each well on a 96-well plate, focused on the cell monolayer surface and captured images [4]. A minimum cell monolayer area of 0.3 mm^2^ per well was investigated. All 150,000 images were visually analyzed for *E*. *coli* pattern formation. Isolates whose images resembled those of the negative control (cells without bacterial incubation) were defined as non-adherent.

## Competing interests

Dirk Roggenbuck is a shareholder of GA Generic Assays GmbH and Medipan GmbH. The remaining authors have no competing as well as non-financial interests.

## Authors’ contribution

UF carried out the *in silico* and *in vitro* studies, evaluated the images, analyzed the data, and drafted and wrote the manuscript. AB and JN programmed the software for VideoScan. JW, JG, and SR participated in the *in vitro* analysis and in technical support. TW provided the human isolates; HA and OZ provided *E. coli* isolates from animals. CS and DR supported the work with scientific advice. PS supervised the work, managed the collection of isolates, wrote and revised the manuscript. All authors read and approved the final manuscript.
